# Expression of the Multimeric and Highly Immunogenic *Brucella* spp. Lumazine Synthase Fused to Bovine Rotavirus VP8d as a Scaffold for Antigen Production in Tobacco Chloroplasts

**DOI:** 10.3389/fpls.2015.01170

**Published:** 2015-12-23

**Authors:** E. Federico Alfano, Ezequiel M. Lentz, Demian Bellido, María J. Dus Santos, Fernando A. Goldbaum, Andrés Wigdorovitz, Fernando F. Bravo-Almonacid

**Affiliations:** ^1^Laboratorio de Virología y Biotecnología Vegetal, INGEBI-CONICET Ciudad Autónoma deBuenos Aires, Argentina; ^2^Instituto de Virología, CICV y A, INTA CastelarBuenos Aires, Argentina; ^3^Fundación Instituto Leloir e Instituto de Investigaciones Bioquímicas de Buenos Aires (IIBBA-CONICET) Ciudad Autónoma deBuenos Aires, Argentina; ^4^Departamento de Ciencia y Tecnología, Universidad Nacional de Quilmes, BernalBuenos Aires, Argentina

**Keywords:** bovine rotavirus, transplastomic plants, tobacco, BLS, VP8, vaccine, lyophilized, IgY

## Abstract

Lumazine synthase from *Brucella* spp. (BLS) is a highly immunogenic decameric protein which can accommodate foreign polypeptides or protein domains fused to its N-termini, markedly increasing their immunogenicity. The inner core domain (VP8d) of VP8 spike protein from bovine rotavirus is responsible for viral adhesion to sialic acid residues and infection. It also displays neutralizing epitopes, making it a good candidate for vaccination. In this work, the BLS scaffold was assessed for the first time in plants for recombinant vaccine development by N-terminally fusing BLS to VP8d and expressing the resulting fusion (BLSVP8d) in tobacco chloroplasts. Transplastomic plants were obtained and characterized by Southern, northern and western blot. BLSVP8d was highly expressed, representing 40% of total soluble protein (4.85 mg/g fresh tissue). BLSVP8d remained soluble and stable during all stages of plant development and even in lyophilized leaves stored at room temperature. Soluble protein extracts from fresh and lyophilized leaves were able to induce specific neutralizing IgY antibodies in a laying hen model. This work presents BLS as an interesting platform for highly immunogenic injectable, or even oral, subunit vaccines. Lyophilization of transplastomic leaves expressing stable antigenic fusions to BLS would further reduce costs and simplify downstream processing, purification and storage, allowing for more practical vaccines.

## Introduction

Rotavirus is the main cause of severe gastroenteritis in newborn mammals and constitutes severe economic losses across the world (Saif and Fernandez, 1996). According to a ten year study spanning from 1994 to 2003, group A bovine rotavirus (BRV) is the main cause of neonatal diarrhea in calves in Argentina, being responsible for substantial economic constraints concerning beef and dairy herd (Garaicoechea et al., 2006). Calves are particularly susceptible to rotavirus infection during the first weeks of life and for that reason it becomes essential to count with an effective system of passive immunization comprising transmission of colostrum and milk antibodies originated from active vaccination of the mothers (Fernandez et al., 1998).

Bovine rotavirus is composed of 11 segments of double-stranded RNA enclosed in an inner core, surrounded by two protein layers that constitute the viral capsid. The outer layer is in turn formed by two different proteins, VP7 and VP4 (Estes et al., 2001). The latter possess hemagglutinating activity and participates in viral adsorption (Kalica et al., 1983; Crawford et al., 1994). VP4 is further cleaved into VP8 and VP5 by proteolytic enzymes in the gastrointestinal tract, being the non-glycosylated VP8 domain the one responsible for viral adhesion to sialic acid residues on the host cells. VP8 also displays neutralizing epitopes which makes it a good candidate for recombinant vaccines (Ruggeri and Greenberg, 1991).

Plant molecular farming offers a low-cost and easy to scale-up alternative for production of recombinant proteins. Plastid genome transformation offers interesting advantages over nuclear transformation for the development of commercial vaccines. Most importantly, the resulting transplastomic plants often present expression levels which are considerably higher than those typically obtained from nuclear transgenic plants. This is a consequence of the polyploidy of the chloroplast genome and the lack of silencing or position effects since the integration events proceed by homologous recombination (Bock, 2007). Furthermore, since chloroplast are maternally inherited in most crops, transplastomic plants imply a lower risk of horizontal gene transfer (Ruf et al., 2007). Particularly, plastid transformation has been extensively involved in the production of antigens, a large number of them are of interest for veterinary medicine (Ruiz et al., 2015).

Lyophilization of transplastomic leaves provides additional benefits which further reduce costs and simplify processing, purification, storage and immunization. It can further concentrate the recombinant products, which can be stable (preserving proper folding and disulfide bond formation) even after prolonged storage at room temperature, thus eliminating the need for cold chain and facilitating transportation (Su et al., 2015). Furthermore, lyophilization also removes microbial contamination from fresh leaves, making oral delivery with powdered lyophilized material in capsules safer (Kwon et al., 2013a).

The C486 BRV VP8^∗^ protein was previously expressed in tobacco chloroplasts, mostly as insoluble aggregates, and it has already been demonstrated that it conferred a strong immune response in female mice. Moreover, suckling mice born to immunized dams were protected against oral challenge with virulent rotavirus (Lentz et al., 2011).

The enzyme lumazine synthase from *Brucella* spp. (BLS) has been described as a novel protein carrier for antigen delivery based on its remarkable physicochemical and immunogenic properties. BLS folds as a highly stable dimer of pentamers, each subunit exposing a N-terminus which can accommodate foreign polypeptides or protein domains without affecting the folding and stability of the decamer as a whole (Zylberman et al., 2004). This characteristic was confirmed by structural analyses of the resulting chimeras. BLS overall structure and characteristics resemble those of the highly stable and immunogenic B subunit of the cholera toxin (CTB) and the *Escherichia coli* heat-labile enterotoxin subunit B (LTB), both of which have been broadly used as potent adjuvants to boost immunogenic response when coupled with foreign antigens. In fact, both adjuvants were already successfully expressed as antigenic fusions in transplastomic plants (Waheed et al., 2011; Lakshmi et al., 2013). BLS also behaves as a potent immunomodulator (Velikovsky et al., 2002; Berguer et al., 2006). It is capable of inducing both humoral and cell-mediated responses (Velikovsky et al., 2003). It should be noted that BLS immunogenic properties only become evident when it is expressed as a fusion and not when it is co-expressed along with the antigen (Craig et al., 2005). Owing to its multivalence and immunogenic properties, BLS was successfully proven as an efficient carrier and adjuvant both for systemic and oral immunization (Rosas et al., 2006). More specifically, the N-terminal end of BLS was fused to the inner domain of the VP8 protein (VP8d) and expressed in *E. coli*. The resulting fusion (BLSVP8d) folded and assembled properly and elicited higher antibody titers than VP8d alone or a mixture of VP8d and BLS, both in female mice and in laying hen models. In the first case, suckling mice born to immunized dams were also protected against oral challenge with virulent rotavirus (Bellido et al., 2009), while immunoglobulin Y (IgY) antibodies against BLSVP8d produced in hens were able to fully protect mice against oral challenge with virulent BRV in a dose-dependent-manner (Bellido et al., 2012).

In this work, and for the first time, the BLS scaffold was assessed in a recombinant plant system. We expressed the BLSVP8d fusion in tobacco (*Nicotiana tabacum L. cv.* Petit Havana) chloroplasts to evaluate the possible use of this antigen for vaccine development. Its immunogenicity was assessed in a laying hen model. Our results demonstrate that BLSVP8d fusion readily accumulated, was highly stable without significant proteolytic degradation and was expressed in soluble form at very high levels in transplastomic leaves. Moreover, the fusion protein remained stable and could be detected in its soluble form in high levels in lyophilized leaves, even after one month storage at room temperature. Unpurified fresh and lyophilized leaf soluble protein extracts were able to induce specific neutralizing IgY antibodies in egg yolk. This work presents an interesting platform for a highly immunogenic injectable, or even oral, VP8^∗^ subunit vaccine. Our results also provide the basis for expression of other subset of antigens in transplastomic plants making use of the highly immunogenic BLS antigen scaffold. Furthermore, lyophilization of transplastomic leaves expressing stable antigenic fusions to BLS would facilitate the processing, purification and storage, reducing costs and allowing for more practical vaccines.

## Materials and Methods

### Chloroplast Transformation Vector Construction

The DNA fragment coding for BLSVP8d, in which VP8d is fused to the N-termini of BLS, was obtained from pET-BLS-VP8d (Bellido et al., 2009) by PCR with *Pfu* DNA polymerase (Invitrogen, Carlsbad, USA) using primers BLSVP8dNdeI (5′ CCCATATGCATGAACCAGTGCTTG 3′) and BLSVP8dXbaI (5′ CCTCTAGATCAGACAAGCGCGATGC 3′). Primers included NdeI and XbaI restriction sites to allow further cloning. The amplification product was subcloned into pZErO-2 (Invitrogen Life Technologies, Carlsbad, SD, USA) and sequenced. BLSVP8d was released by enzymatic digestion with NdeI and XbaI, gel purified and cloned into chloroplast transformation vector pBSW-utr/hEGF (Wirth et al., 2006), which was previously digested with NdeI and XbaI to excise the hEGF sequence. The resulting construction was named pBSW-utr/BLSVP8d and carries the BLSVP8d sequence under the control of the promoter and 5′-untranslated region of the tobacco *psbA* gene (5′*psbA*) and downstream the *aadA* sequence that confers spectinomycin resistance, under the transcriptional control of the *rrn* promoter (Prrn). Flanking regions were included to allow homologous recombination with *N. tabacum* plastome (GenBank accession number NC 001879). The left flanking region (LFR) included the 3′ region of *rrn16*, and the right flanking region (RFR) contained the full *trnI* and the 5′region of *trnA*, all from *N. tabacum*.

### Chloroplast Transformation and Molecular Characterization of Transplastomic Plants

Chloroplast transformation was carried out as previously described (Svab et al., 1990), using a PDS 1000/He biolistic device (Bio-Rad, USA). Fully expanded leaves of *in vitro* cultured *N. tabacum* cv. Petit Havana plants were bombarded with 50 mg of 0.6 μm gold particles (Bio-Rad) coated with 2 μg of plasmid DNA, using 1,100 psi rupture disks (Bio-Rad). Transformed shoots were regenerated in selective RMOP regeneration medium containing 500 mg/l spectinomycin dihydro-chloride. To obtain homoplastic plants, leaves from PCR-positive shoots were cut into pieces and taken through two additional regeneration cycles in selective medium. After rooting, plants were transferred to soil and grown under greenhouse conditions. In the greenhouse, natural light was supplemented 16 h/day by sodium lamps providing 100–300 μmol s^-1^ m^-2^, temperature was set at 26°C during day and 19°C in the night.

### PCR Analysis to Confirm Transplastomic State

DNA obtained from leaf material of spectinomycin resistant and wild-type (wt) plants was used as template for amplification with primers Cl Fw (5′-GTATCTGGGGAATAAGCATCGG-3′) and Cl Rev (5′- CGATGACGCCAACTACCTCTG-3′). Cl Fw hybridizes upstream of the LFR to 16S wt gene and Cl Rev hybridizes to the *aadA* sequence. Therefore, a 1450 bp fragment was only amplified from transplastomic plants.

The PCR reaction was conducted in a total volume of 50 μl, containing 10 ng of leaf total DNA, 10X PCR buffer, 400 μM dNTP mix, 150 ng of each primer, and 1 U *Taq* polymerase (Invitrogen Corp., Carlsbad, SD, USA). The reaction conditions were as follows: initial denaturalization (95°C, 5 min) was followed by 30 amplification cycles (denaturing, 95°C, 30 s; annealing, 55°C, 60 s, and extension 72°C, 90 s) and a 10 min final extension step at 72°C.

### Southern Blot

Total DNA was extracted from leaves as described by Dellaporta et al. (1984). The DNA (2.5 μg) was digested overnight with NcoI enzyme (New England Biolabs, USA), electrophoresed in 0.8% agarose gels and blotted onto Hybond-N+ Nylon membranes (Amersham Biosciences, USA). Specific DNA sequences were detected by hybridization with α-^32^P-labeled *trnI/A* DNA probe. The probe was generated by random priming with a Prime-a-Gene kit (Promega, USA). Pre-hybridization and hybridization were carried out at 65°C in Church’s hybridization solution (Church and Gilbert, 1984) for 2 and 16 h, respectively. Membranes were washed twice with gentle shaking for 30 min in 0.2X SSC, 0.1% SDS at 65°C. The blot was exposed to a storage phosphor screen, which was analyzed in a Storm 840 PhosphorImager system (Amersham).

### Northern Blot

Total RNA was extracted from fully expanded young leaves using TRiZOL Reagent (Invitrogen Corp., Carlsbad, SD, USA). An aliquot of 5 μg of formaldehyde-denatured RNA was electrophoresed in a 1.5% agarose/formaldehyde gel and blotted onto Hybond-N+ Nylon membranes (Amersham Biosciences). Specific mRNA sequences were detected by hybridization with α-^32^P-labeled *bls* DNA probe generated by random priming with a Prime-a-Gene kit (Promega). The blot was pre-hybridized, hybridized and washed as described for Southern blot.

### Total Protein Analysis

Total protein content from transformed and non-transformed plants was extracted by grinding 25 mg of fresh leaf material in 125 μl of Laemmli buffer. Different quantities of samples were electrophoresed in 12.5% SDS-PAGE gels for either Coomassie blue staining or western blotting. For western blot, proteins were transferred onto a nitrocellulose membrane. The membrane was probed with mouse antiserum against VP8 or BLS, followed by three washes with 50 mM Tris-HCl, pH 8, 150 mM NaCl, 0.05% Tween 20, and a second incubation step with an alkaline phosphatase-linked goat anti-mouse IgG antibody diluted to 1:2000. Finally, phosphatase activity was determined by a chromogenic reaction by addition of NBT/BCIP (Nitro blue tetrazolium/5-Bromo-4-chloro-3-indolyl phosphate; Sigma Chemical Co., USA) as substrates.

### BLSVP8d Solubility Analysis and Quantification

Total protein from 200 mg of fully expanded leaves from transformed and non-transformed plants was extracted in 1 ml of protein extraction buffer (50 mM Tris–HCl, pH 8, 30 mM NaCl). For lyophilized leaves, approximately 1/10 of leaf was added per ml of extraction buffer. Briefly, leaf material was ground in liquid nitrogen, mixed with extraction buffer and sonicated three times for 15 s with a microtip (Heat System Ultrasonic, Farmingdale, NY, USA) set at level 50%, and then centrifuged for 15 min at 10,000 × *g*, 4°C. The supernatant and pellet fractions contained soluble and insoluble proteins, respectively. The pellet was washed three times with 1 ml water at 0°C. Samples from each fraction (total, soluble, insoluble) were mixed with Laemmli buffer and were electrophoresed in 12.5% SDS-PAGE for either Coomassie blue staining or western blotting. Total soluble protein (TSP) content from supernatant fractions was quantified by Bradford assay using bovine serum albumin (BSA; Sigma Aldrich) as standard. For recombinant protein level quantification, gels were scanned and protein bands were quantified using Image J software (NIH, http://rsbweb.nih.gov/ij). For western blot, proteins were transferred onto nitrocellulose membrane. Membranes were treated as described before for total protein analysis.

### Lyophilization

Fully expanded leaves from transformed and non-transformed plants were cut into small fragments measuring roughly 1 cm^2^. Fragments were frozen at -80°C and then lyophilized in a Freezone Lyph-Lock 6 Freeze Dry System (Labconco) in vacuum (0.13 mBar) for at least 1 day at -40°C. Lyophilized samples were stored at -80°C or at room temperature.

### Animal Immunization

Soluble quantified extracts from either fresh or lyophilized leaves from transformed and non-transformed plants were used for immunization of groups of light brown laying hens (*n* = 2) seronegatives for BRV. Groups were inoculated with 3.83 μg of BLSVP8d of which 2 μg corresponded to VP8d. Recombinant fresh and lyophilized extracts were normalized with the corresponding non-transformed extracts. Hens received three intramuscular doses of 0.5 ml, on days 0, 30, and 75, consisting of Freund’s incomplete adjuvant:antigen in a proportion of 50:50. Laying capacity and the sites of injection were checked for side effects. Eggs were collected on day 0 and then weekly, 15 days after the third immunization, on days 87, 94, and 103 (DPI).

Hens were obtained from the biotery of the CICVyA, INTA. Animal immunization, management and sample collection were conducted by trained personnel under the supervision of a veterinarian, in accordance with protocols approved by INTA’s ethical committee of animal welfare.

### Antibody Measurements by ELISA

The egg yolk purification protocol was adapted from Akita and Nakai (1993). Egg yolks were diluted with five volumes of distilled water, frozen at -80°C, thawed on ice and centrifuged at 4°C for 10 min at 8,000 × *g*. The supernatant was stored at 4°C and used for IgY analysis. Titers of IgY against BLSVP8d were determined according to Bellido et al. (2012). Briefly, 1 μg of purified VP8d was directly adsorbed onto each well overnight in carbonate-bicarbonate buffer pH 9.6 and blocked with PBS-T, 5% normal horse serum for 1 h at 37°C. Blocking buffer was discarded and fourfold dilutions, starting at a 1/20 dilution, of all IgY samples in blocking buffer were incubated for 1 h at 37°C. Wells were then washed with PBS-T and incubated with horseradish peroxidase-labeled goat anti-chicken IgY (Sigma Chemical Co., USA). After thorough washing, the reaction was developed with ABTS/H_2_O_2_ system and stopped by addition of 5% SDS. Absorbance was measured at 405 nm (A405) in a Multiskan Ex, Labsystems Inc. The cut-off value of the assay was calculated as the mean specific optical density (OD) plus 3 standard deviations from IgY purified samples obtained from hens immunized with soluble wild-type plant extracts. A previously purified IgY against BLSVP8d expressed in *E. coli* was used as a positive control.

Titers were expressed as the reciprocal of the highest IgY dilution which presented an OD value above the cut-off value.

### Viral Neutralization Assay

Virus-neutralizing titers were determined by a fluorescent focus reduction test as previously described (To et al., 1998). Briefly, serial diluted samples of IgY antibody fractions were mixed with an equal volume of C486 strain BRV in order to have 100 fluorescent focus-forming (FFU)/100 μL of mixture that was incubated for 1 h at 37°C. Then they were inoculated onto MA-104 monolayers (four replicates) and were further incubated for 48 h at 37°C. Plates were then fixed with 70% acetone and were developed using a fluorescein isothiocyanate-labeled anti-BRV polyclonal antibody derived from the hyperimmunization of a colostrum-deprived calf. Samples that generated >80% reduction of the infection rate were considered protective. The viral neutralization titer was calculated by the Reed and Muench (1938) method considering the highest sample dilution that resulted positive.

## Results

### Production of Transplastomic *N. tabacum* Expressing BLSVP8d

**FIGURE 1:**
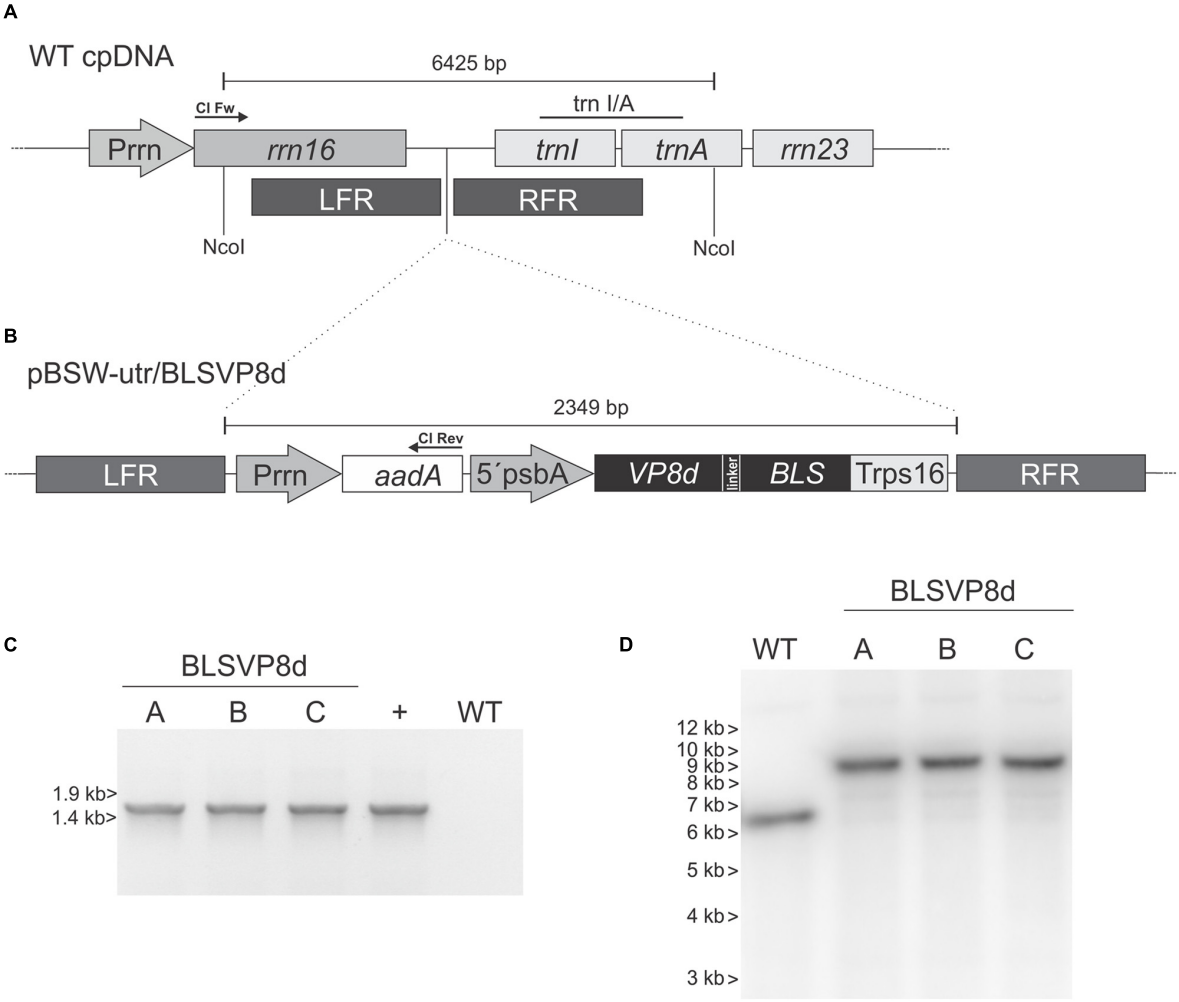
**Chloroplast transformation vector and analysis of transgene integration.** **(A)** Chloroplast genome (WT cpDNA) physical map at the insertion site (dotted lines) showing the left and right flanking recombinogenic regions (LFR and RFR). **(B)** Vector pBSW-utr/BLSVP8d contains VP8d from BRV strain C486 sequence fused to *Brucella* spp. lumazine synthase (BLS) under the 5′ untranslated sequence and the promoter of the *psbA* gene (5′psbA). The LFR includes the 3′ region of *rrn16*, and the RFR contains the full *trnI* and the 5′region of trnA. The *aadA* sequence is under the control of the Prrn promoter. *rrn23*: sequence coding the 23S rRNA (Wirth et al., 2006). *trnI/A*: probe used in Southern blot assays. Arrows indicate the location of the forward (Cl Fw) and reverse (Cl Rev) primers used for the PCR analysis. **(C)** PCR analysis to confirm integration of the recombinant vector into the wild-type plastome. WT: wild-type tobacco plant. The plasmid used for plant transformation was included as a positive control (+). The position of Lambda BstEII marker is indicated on the left. **(D)** Southern blot using *trnI/A* probe to confirm integration and homoplasmy (wild-type plastome: 6.4 kbp, transformed plastome: 8.8 kbp). The position of 1-kb DNA marker is indicated on the left. (A-C) independent BLSVP8d transplastomic lines.

Expression from chloroplast transformation vector pBSW-utr/BLSVP8d (**Figures 1A,B**) was first assessed in *Escherichia coli* extracts since the prokaryotic protein synthesis machinery can recognize plastid transcriptional and translational elements. *E. coli* does not necessarily reflect the environment of the chloroplast stroma, nevertheless it provides a fast method for testing the final genetic construction. The recombinant protein accumulated to high levels in bacteria, being detectable both by SDS-PAGE followed by Coomassie blue staining and western blot (data not shown).

Chloroplasts from *in vitro* tobacco plants (*N. tabacum* L. cv. Petite Havana) were then transformed by particle bombardment using vector pBSW-utr/BLSVP8d. Several regenerating shoots were obtained and three of them representing independent plastid-transformed (transplastomic) lines were further confirmed by PCR (**Figure 1C**). These lines were subjected to two additional rounds of regeneration on spectinomycin supplemented media to achieve homoplasmy. Rooted plants were transferred to soil and further grown under greenhouse conditions. Surface sterilized seeds from these plants were germinated in spectinomycin containing media to confirm maternal inheritance of the transgenes and the absence of wild-type plastomes. Germinated plants were transferred to greenhouse for further analysis. Phenotypic comparison to wild-type plants revealed that they are indistinguishable from their non-transformed counterparts (**Supplementary Figure S1**).

### Analysis of Transgene Integration and Homoplastic State

Southern blot was performed in order to evaluate stable integration into the plastome and homoplasmy of spectinomycin resistant lines germinated from seeds. For this purpose total leaf DNA was digested with NcoI, whose recognition sequence is absent in the integrated cassette of transplastomic plants and which cuts both upstream and downstream of the integration site in the plastome. Therefore, bands of approximately 6.4 and 8.8 kbp are expected for wild-type and transplastomic plastomes, respectively, when using a ^32^P-labeled probe comprising a fragment from *trnI/trnA*. Southern blot analysis confirmed the integration for all the evaluated lines (**Figure 1D**).

### Analysis of Transcripts

**FIGURE 2:**
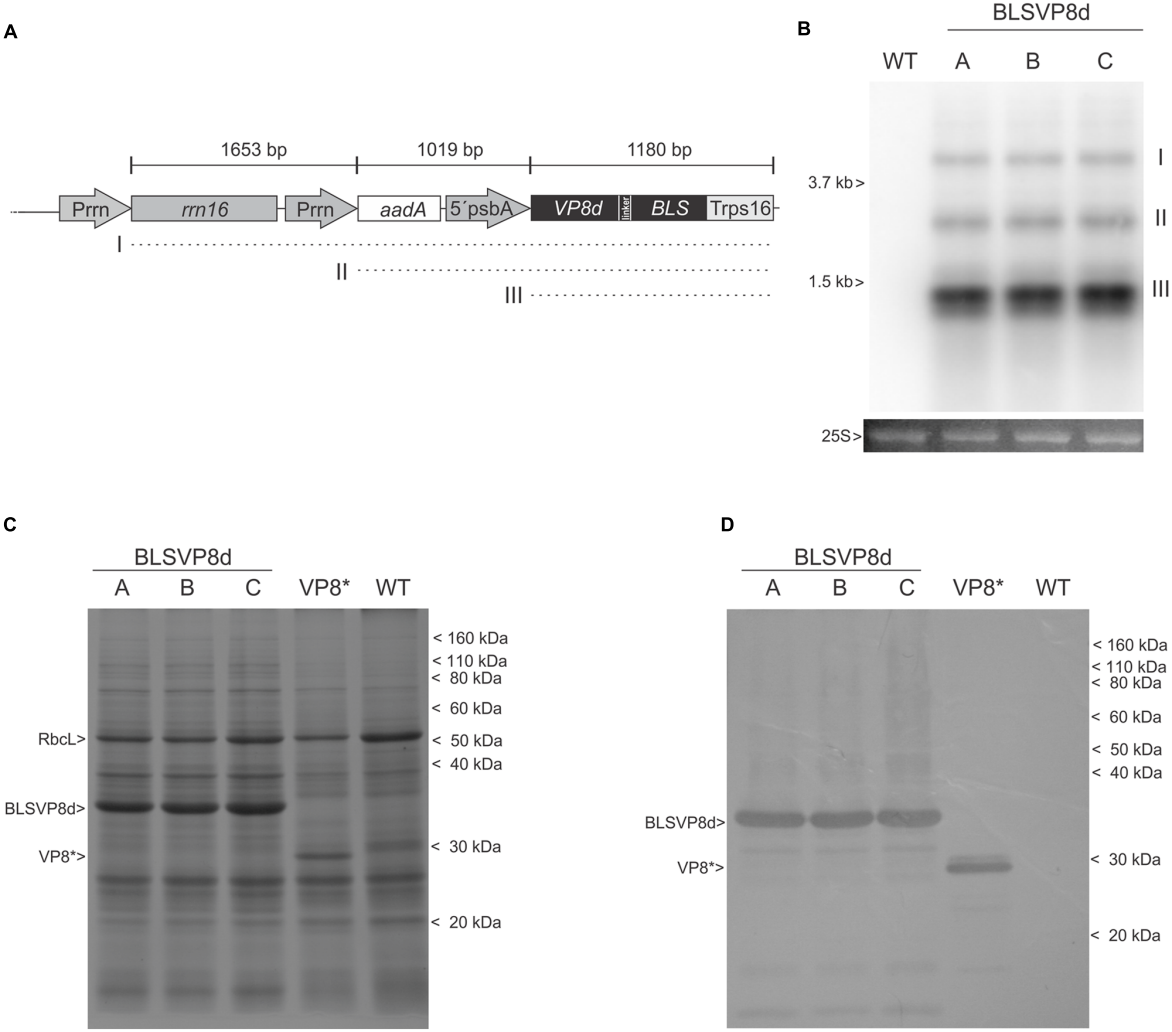
**Characterization of transcripts and BLSVP8d expression.** **(A)** Physical map showing expected transcripts generation from the integrated cassette. (I) polycistronic read-through transcript (3.8 kb) (II) biscistronic transcript (2.2 kb). (III) monocistronic transcript (1.2 kb). **(B)** Northern blot using *bls* probe showing transcript generation. Transcripts are shown relative to 25S and 16S ribosomal RNA (3.7 and 1.5 kb, respectively). The 25S rRNA is shown as total RNA lane load control. **(C)** 12.5% SDS-PAGE gel stained with Coomassie brilliant blue and **(D)** western blot using an antiserum against VP8^∗^. Total protein from 3 mg of transplastomic WT, BLSVP8d, and VP8^∗^ leaves was extracted in Laemmli buffer. Transplastomic VP8^∗^ extract was included as a positive control. The position of molecular weight marker (Novex Sharp Pre-Stained Protein Standard) is shown on the right. BLSVP8d (35,5 KDa), VP8^∗^ (31 KDa), and RuBisCo large subunit (RbcL) positions are shown on the left. (A-C) independent BLSVP8d transplastomic lines.

Northern blot was performed to evaluate the presence of transcripts containing the BLSVP8d sequence. Total leaf RNA was probed with a sequence homologous to *bls* and as a result three major transcripts could be detected in all transplastomic lines. The strong promoter *psbA* accounted for the smallest and most abundant monocistronic transcript, the *rrn* promoter included in the cassette controlling *aadA* and BLSVP8d expression was responsible for a bicistronic transcript and a larger transcript aroused from read-through transcription from an endogenous *rrn* promoter (**Figures 2A,B**). Expected transcripts were approximately 1.2 kb for the monocistron, 2.2 kb for the bicistron and 3.8 kbp for the larger one. This interpretation is supported by comparing the electrophoretic mobility of these three transcripts relative to the one of rRNA 25S and 16S (3.7 and 1.5 kb, respectively).

### BLSVP8d Expression and Stability in Fresh Leaves

Expression of BLSVP8d was assessed in total protein extracts of all transplastomic lines. An intense differential band of approximately 35 kDa and compatible with BLSVP8d expected molecular weight was evident for the three independent lines and absent in the wild-type control after Coomassie blue staining of a SDS-PAGE. Its identity was further confirmed by western blot using an antiserum raised against a larger version of VP8d produced in *E. coli* (VP8^∗^) (Bellido et al., 2009). A total extract from a transplastomic plant expressing VP8^∗^ which was previously generated at our lab was included as a positive control (Lentz et al., 2011). Remarkably, the band corresponding to BLSVP8d was even more intense than that of Rubisco large subunit (RuBisCo; **Figures 2C,D**).

**FIGURE 3:**
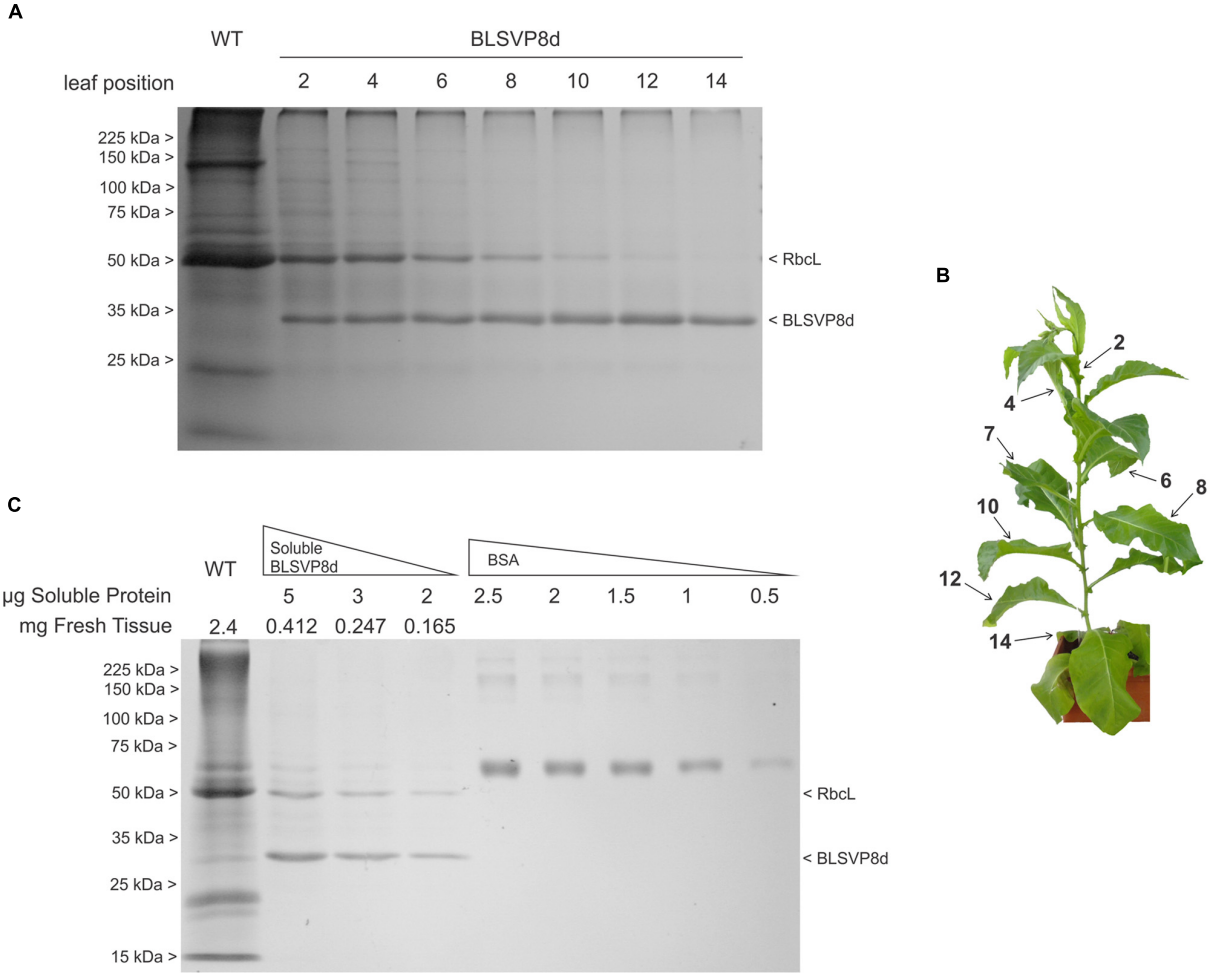
**BLSVP8d accumulation along development and quantification of soluble BLSVP8d in fresh leaf.** **(A)** 12.5% SDS-PAGE gel stained with Coomassie brilliant blue. Total protein was extracted in Laemmli buffer from 2 mg of different leaves along a BLSVP8d plant. Positions of molecular weight marker (Broad Range Protein Molecular Weight Markers), BLSVP8d and RbcL are indicated on the margins. **(B)** Transplastomic BLSVP8d plant used in the stability assay. Leaves were numbered from the first expanded leaf from the top to the bottom. **(C)** 12.5% SDS-PAGE gel stained with Coomassie brilliant blue showing different quantities of total soluble protein from an intermediate transplastomic BLSVP8d leaf (leaf 7). BLSVP8d was quantified against a calibration curve of Bovine Serum Albumin (BSA). A total wild-type extract was used as control. Protein extract quantities, RbcL, BLSVP8d, and molecular weight marker (Broad Range Protein Molecular Weight Markers) positions are indicated on the margins.

Age-related leaf protein content decline can be exploited to evaluate recombinant protein stability (Birch-Machin et al., 2004; Zhou et al., 2008). In order to evaluate BLSVP8d stability, total protein extracts from leaves along a transplastomic line were examined by Coomassie blue staining (**Figures 3A,B**). BLSVP8d fusion readily accumulated in younger leaves and it remained highly stable in mature and even in senescent leaves, without showing any important signs of proteolysis. The highest expression levels were observed in old leaves. In contrast, RuBisCo large subunit and the majority of other proteins were gradually degraded due to senescence mechanisms. BLSVP8d levels were similar to, or greater than, those of RuBisCo depending on the leaf selected (**Figures 3A,B**).

### BLSVP8d Quantification in Fresh Leaf

A mature leaf was chosen, total protein content was determined by Bradford and BLSVP8d was quantified by densitometry against a BSA standard by SDS-PAGE followed by Coomassie blue staining. BLSVP8d expression accounted for, at least, 40% of TSP or 4.85 mg/g fresh tissue, which represents roughly 10 times the previously observed levels for VP8^∗^ (**Figure 3C**).

### Stability and Solubility in Fresh and Lyophilized Leaf Material

**FIGURE 4:**
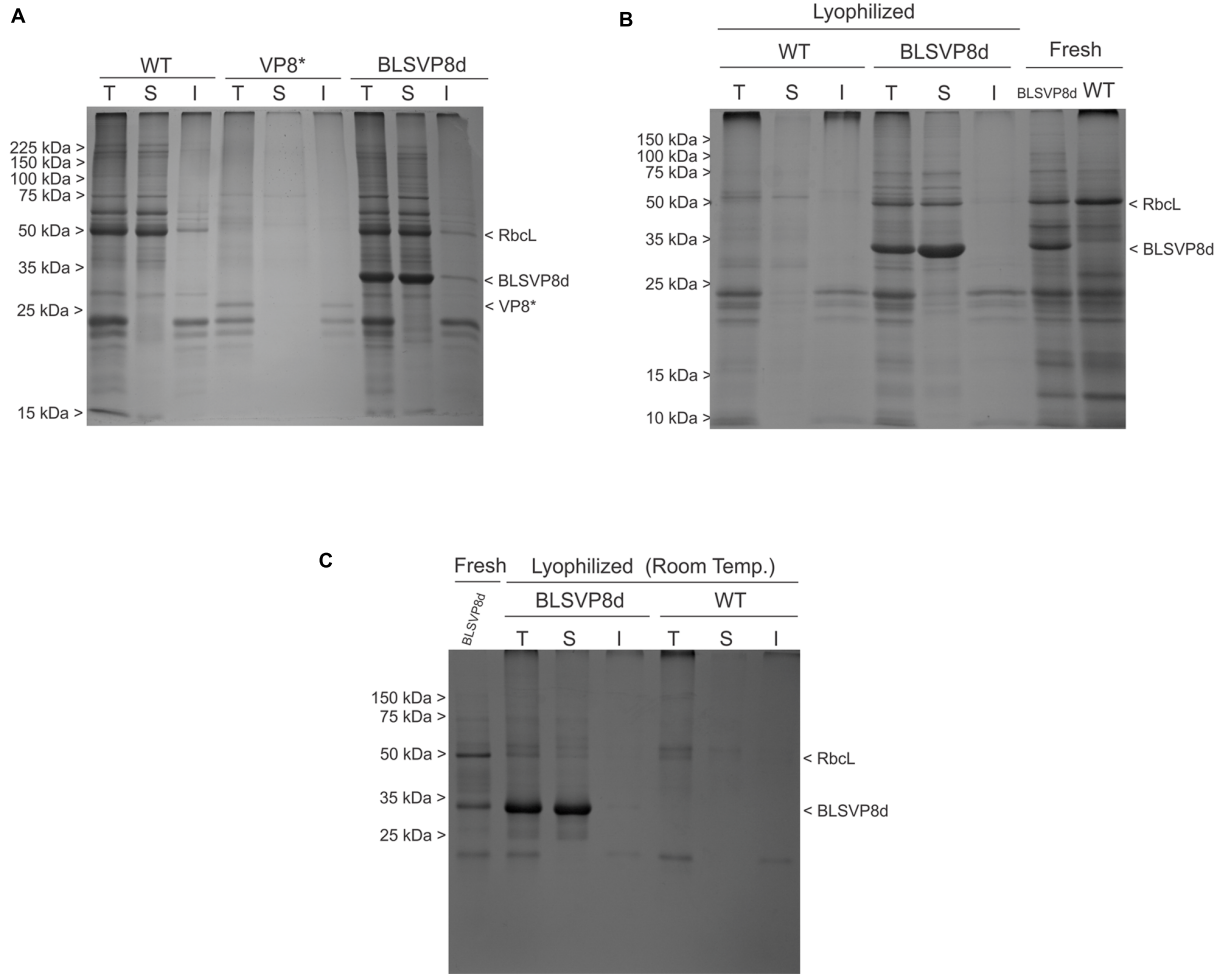
**BLSVP8d solubility and stability in fresh and lyophilized leaves.** 12.5% SDS-PAGE gels stained with Coomassie brilliant blue showing total (T), soluble (S), and insoluble (I) protein fractions from: **(A)** 2 mg of transplastomic WT, BLSVP8d, and VP8^∗^ leaves extracted in Laemmli buffer. VP8^∗^ was included as an insoluble control. **(B)** 0.25 mg of transplastomic WT and BLSVP8d leaves from lyophilized leaves stored at -80°C and extracted in Laemmli buffer. Total extracts (3 mg) from wild-type and BLSVP8d fresh leaves were included as controls. **(C)** 0.25 mg of lyophilized leaves stored at room temperature for one month and extracted in Laemmli buffer. A total extract (2 mg) from a BLSVP8d fresh leaf was included as control. BLSVP8d, VP8^∗^, RbcL, and molecular weight marker (Broad Range Protein Molecular Weight Markers) positions are indicated on the margins.

In a preliminary analysis, both VP8^∗^ and BLSVP8d proved to be insoluble when expressed from their respective chloroplast transformation vectors in *E. coli*. Since the majority of VP8^∗^ also accumulated as inclusion bodies in the chloroplast stroma and several sonication pulses were necessary in order to have it extracted in a saline buffer compatible with immunization studies (Lentz et al., 2011), BLSVP8d solubility in fresh leaves was assayed. Therefore, leaf extracts from wild-type, BLSVP8d and VP8^∗^ plants containing total (T), soluble (S), and insoluble (I) protein were compared by SDS-PAGE followed by Coomassie blue staining. As expected, VP8^∗^ was mostly insoluble. Surprisingly, BLSVP8d mainly remained soluble in fresh leaf chloroplasts (**Figure 4A**). Identity of recombinant protein bands was confirmed by western blot (not shown).

Extracts from lyophilized transplastomic and wild-type leaves were analyzed in parallel by SDS-PAGE followed by Coomassie blue staining. BLSVP8d fusion remained stable and soluble even after lyophilization (**Figure 4B**). Comparing these extracts to their respective total extracts from fresh leaves, extracts from lyophilized leaves presented more degradation of endogenous proteins, which meant intact BLSVP8d become slightly more concentrated after lyophilization. As a result, soluble BLSVP8d extracts from lyophilized leaves were also included in immunization assays.

The soluble and stable nature of BLSVP8d remained unaltered even after storage for one month at room temperature (**Figure 4C**).

### Immune Response to Transplastomic BLSVP8d

**FIGURE 5:**
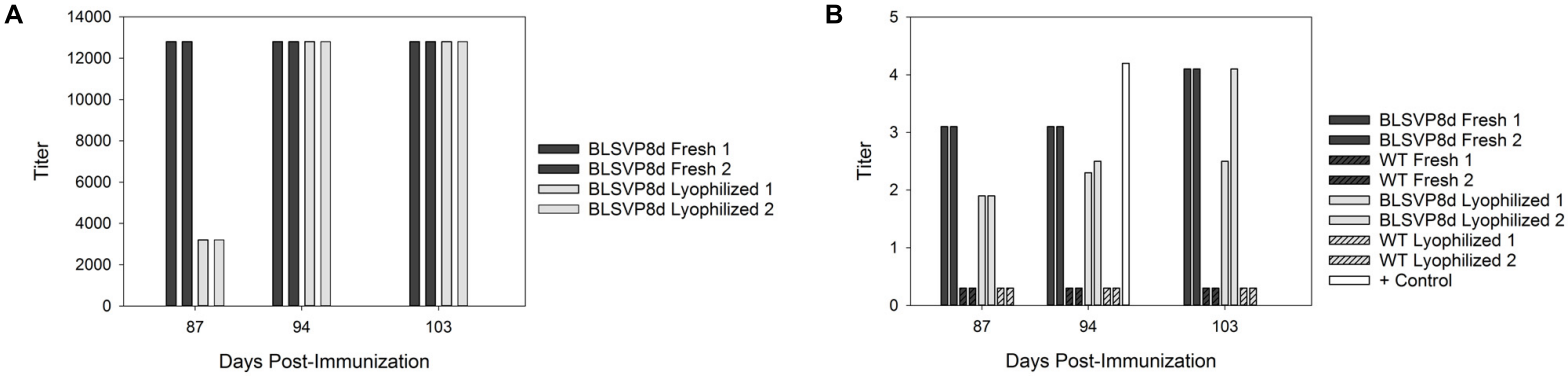
**Detection of specific neutralizing immunoglobulin Y (IgY) antibodies against BLSVP8d after immunization of hens with either fresh or lyophilized transplastomic soluble protein extracts.** **(A)** Detection of specific IgY antibodies in egg yolk obtained from hens immunized with total soluble protein extracts. Titers are indicated both for fresh (dark bars) and lyophilized (light bars) samples. Animals received three doses containing 3.83 μg of BLSVP8d (2 μg corresponded to VP8d). As a control, groups of hens were also immunized with total soluble protein extracts obtained from fresh and lyophilized wild-type leaves. Purified IgY response was evaluated by ELISA. Titers were expressed as the reciprocal of the highest IgY dilution which gave an OD value above the mean values of the respective control samples plus 3 standard deviations. **(B)** Viral neutralization assay to assess the prevention of bovine rotavirus infection *in vitro* conferred by specific IgY antibodies. Titers were expressed by the Reed and Muench’s (1938) method considering the highest dilution that resulted in an 80% reduction of fluorescent foci. Titers are indicated both for fresh (dark bars) and lyophilized (light bars) samples. An IgY fraction obtained by immunization of a hen with BLSVP8d previously produced in *E. coli* was included as control (white bar).

Immunoglobulin Y antibodies specific to BLSVP8d could be detected by ELISA after immunization of hens with either fresh or lyophilized transplastomic soluble protein extracts. No specific antibodies could be detected after immunization with the respective wild-type extracts. Overall, the response was slightly faster for fresh extracts. Antibody titers reached 12800 and ranged between 3200 and 12800 for fresh and lyophilized extracts, respectively (**Figure 5A**).

A virus neutralization assay was performed to assess the ability of these specific IgY antibodies to prevent bovine rotavirus infection *in vitro*. The anti-BLSVP8d IgY antibodies were able to neutralize rotavirus infection, in accordance with the results previously obtained by ELISA. Titers were similar for IgY antibodies elicited after immunization with both fresh and lyophilized transplastomic soluble protein extracts, and once again the response was slightly faster in the case of fresh leaf extracts. In addition, no neutralizing activity could be detected for any of the IgY fractions obtained after immunization with the respective wild-type fresh or lyophilized soluble protein extracts (**Figure 5B**).

## Discussion

Plastid genome transformation constitutes an advantageous system for recombinant protein expression. Chloroplast molecular farming stands out for many reasons, being of particular interest the reduced manufacturing costs, the ease of scaling-up, the high expression levels that are typically obtained and which simplify and reduce the cost of immunization protocols, the absence of human pathogens or toxins, the post-translational modification capability, the disulfide bond formation and the facilitated biological containment since chloroplasts are not inherited in polen. In the last years, several studies have reported lyophilization of transplastomic leaves expressing vaccine antigens or biopharmaceuticals, further reducing costs and facilitating storage, processing and purification, which makes immunization protocols simpler and cost-effective (Rosales-Mendoza et al., 2009; Soria-Guerra et al., 2009; Manganelli et al., 2012; Cardona et al., 2013; Kwon et al., 2013a,b).

In this work, we report for the first time the production of transplastomic plants expressing a recombinant fusion of BLS, a highly immunogenic decameric protein from *Brucella* spp, to an antigen. We have recently succeeded in producing transplastomic tobacco which expressed BRV C486 VP8^∗^ protein, an interesting target for vaccine development since it is involved in rotavirus infectivity and neutralization. Based on our previous work, BLS was coupled to VP8d, a shorter fragment of VP8^∗^, and transplastomic BLSVP8d plants were obtained. Since properly folded BLSVP8d had already been produced in bacteria and its potent immunomodulatory properties had already been demonstrated in female mice and laying hen models, it was a good candidate to further analyze its immunogenic properties in a recombinant plant system (Bellido et al., 2009, 2012).

Our results show that BLSVP8d was highly expressed in soluble form in plants, accounting for 40% of TSP or 4.85 mg per gram of fresh leaf tissue, which is approximately one order above the level obtained for VP8^∗^ using the same transformation vector. In the same manner as VP8^∗^, BLSVP8d readily accumulated in young leaves and remained stable in mature and senescent leaves of transplastomic plants. Stable BLSVP8d become more evident with leaf age as the content of RuBisCo and other proteins declined, meaning older leaves had greater BLSVP8d expression than the one reported for an intermediate mature leaf. Similar pattern of degradation of protein content was also observed for lyophilized leaves, which could also contribute to additional concentration of intact BLSVP8d. Our group has previously shown the capability of transplastomic tobacco to produce large quantities of immunogenic proteins without any observable phenotypic effects. Such is the case of VP1 peptide from the foot and mouth disease virus (FMDV) fused to the β-glucuronidase enzyme, in which expression levels (51% of TSP) were clearly higher than those of RuBisCo large subunit, the most abundant leaf protein in a wild-type plant (Lentz et al., 2009). Transplastomic BLSVP8d levels were also similar to, or greater than, those of RuBisCo depending on the age of the analyzed leaf. The drastic increase in the expression levels of BLSVP8d compared to VP8^∗^ could not be solely attributed to the fusion to BLS, since VP8d is almost one third smaller having truncated amino and carboxi-termini. Nevertheless, C-terminal fusion to BLS did not appear to be unstabilizing at all in the chloroplast stroma and although proper folding was not established in plants, BLS fusion protein decamers could account for reduced proteolytic degradation and protection. Despite the high expression levels, wild-type and transplastomic BLSVP8d plants were phenotypically indistinguishable from each other.

Since plastids are evolutionarily derived from bacteria, and both share compatible transcriptional and translational machinery, expression analysis from the transformation vector was first assessed in *E. coli*. Nevertheless, accumulation and stability could not be accurately predicted in this prokaryotic model. BLSVP8d was highly expressed in bacteria but formed inclusion bodies. VP8^∗^ was also mainly expressed as inclusion bodies either in bacteria and chloroplasts. Interestingly, despite its higher expression levels, we demonstrated that transplastomic BLSVP8d always remained soluble, even in senescent or lyophilized leaves. Furthermore, storage of lyophilized material for up to one month at room temperature did not show any sign of degradation or altered solubility of BLSVP8d. The observed difference in solubility between VP8^∗^ and BLSVP8d could not be entirely associated with the addition of BLS since VP8^∗^ and VP8d have different length. However, by no means the multimeric nature of BLS was detrimental for the solubility of the fusion as a whole.

Our work also demonstrated that hen immunization with unpurified transplastomic BLSVP8d soluble extracts from both fresh and liophylized leaves induced IgY specific neutralizing antibodies in egg yolk, as measured by ELISA and *in vitro* neutralization assays. Immunization results could not be directly compared to those obtained for VP8^∗^ due to its difference in length, but all our previous results with BLSVP8d expressed in bacteria suggest that potentially enhanced immunogenicity should also be the case in plants. Currently ongoing research is aimed to ascertain this and to assess stability, solubility and proper folding of BLSVP8d multimers coming from lyophilized extracts after longer storage at room temperature. Moreover, dietary uptake of powdered egg yolk specific IgY antibodies could be exploited in the future as an approach to confer passive protection against rotavirus (Vega et al., 2012).

## Conclusion

The BLS scaffold was assessed for the first time in plants. BLSVP8d was highly expressed in tobacco chloroplasts, remaining soluble and stable during all stages of plant development, even in senescent or lyophilized leaves. Furthermore, fresh and lyophilized leaf unpurified soluble extracts were able to induce specific neutralizing IgY antibodies in a laying hen model. This work presents BLS as an interesting platform for a plant-based highly immunogenic injectable, or even oral, VP8^∗^ subunit vaccine.

Our findings provide the basis for the expression of other subset of antigens fused to the potent immunomodulator BLS and suggest that lyophilization, without the need for additional lyoprotective components, of transplastomic leaves expressing antigenic fusions can further reduce costs and simplify downstream processing, purification and storage, allowing for more rational injectable or oral vaccines.

## Author Contributions

EFA was responsible for the acquisition, analysis, and interpretation of the data, the drafting of the manuscript, the publishing approval and accountable for all aspects of the work. EML was responsible for design and critically revision of the work, the publishing approval, and accountable for all aspects of the work. DB was responsible for the acquisition, analysis, and interpretation of the data, the publishing approval and accountable for all aspects of the work. MJDS was responsible for design and critically revision of the work, the publishing approval and accountable for all aspects of the work. FAG was resposible for design and criticaly revision of the work, the publishing approval and accountable for all aspects of the work. AW was resposible for conception and drafting of the work, interpretation of the data, the publishing approval and accountable for all aspects of the work. FBA was resposible for conception and drafting of the work, interpretation of the data, the publishing approval and accountable for all aspects of the work.

## Conflict of Interest Statement

The authors declare that the research was conducted in the absence of any commercial or financial relationships that could be construed as a potential conflict of interest.
