# Supplementary material for: Expression of the Multimeric and Highly Immunogenic Brucella spp. Lumazine Synthase Fused to Bovine Rotavirus VP8d as a Scaffold for Antigen Production in Tobacco Chloroplasts
Source: Front Plant Sci. 2015 Dec 23;6:1170. doi: 10.3389/fpls.2015.01170 (PMC4688359; doi:10.3389/fpls.2015.01170)
Supplement: Supplementary file 1 [file Image_1.PDF]

Supplementary Material

Expression of the multimeric and highly immunogenic *Brucella* spp. lumazine synthase fused to bovine rotavirus VP8d as a scaffold for antigen production in tobacco chloroplasts

E. Federico Alfano, Ezequiel M. Lentz, Demian Bellido, María José Dus Santos, Fernando A. Goldbaum, Andrés Wigdorovitz, Fernando F. Bravo-Almonacid*

*** Correspondence:** Fernando F. Bravo-Almonacid: fbravo@dna.uba.ar

1 Supplementary Figures and Tables

1.1 Supplementary Figures

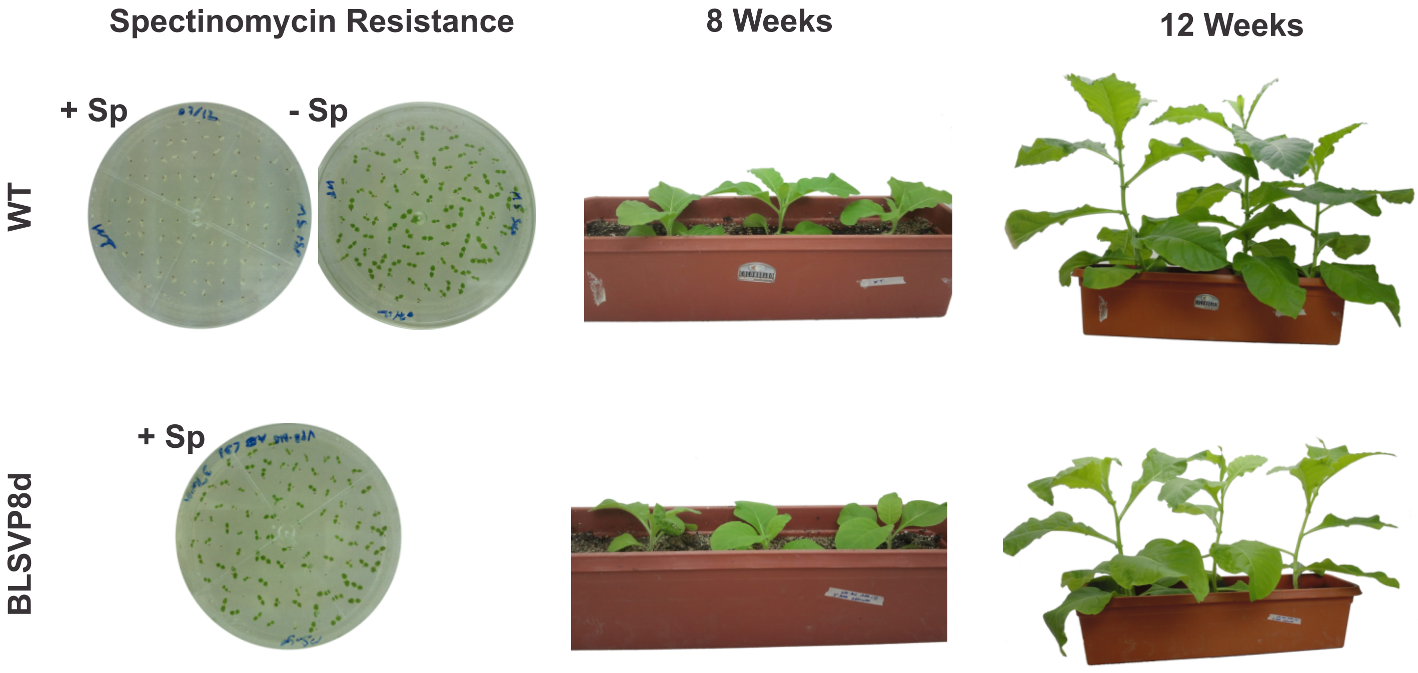

Supplementary Figure 1. Phenotypic characterization of transplastomic *N. tabacum* L. cv. Petit Havana BLSVP8d plants. Seeds from wild-type (WT) or transplastomic plants were germinated in selective media containing spectinomycin (500 mg/L). Phenotypes were registered 10 days after sowing the seeds. Transplastomic plants were then grown under greenhouse conditions and phenotypes were registered 8 and 12 weeks post sowing.
